# Expanding our grasp of two-component signaling in *Clostridioides difficile*

**DOI:** 10.1128/jb.00188-23

**Published:** 2023-09-20

**Authors:** Orlando Berumen Alvarez, Erin B. Purcell

**Affiliations:** 1 Department of Chemistry and Biochemistry, Old Dominion University, Norfolk, Virginia, USA; Ohio State University, Columbus, Ohio, USA

**Keywords:** *Clostridioides difficile*, two-component signaling

## Abstract

The intestinal pathogen *Clostridioides difficile* encodes roughly 50 TCS, but very few have been characterized in terms of their activating signals or their regulatory roles. A. G. Pannullo, B. R. Zbylicki, and C. D. Ellermeier (J Bacteriol 205:e00164-23, 2023, https://doi.org/10.1128/jb.00164-23) have identified both for the novel *C. difficile* TCD DraRS. DraRS responds to antibiotics that target lipid-II molecules in the bacterial cell envelope, and regulates the production of a novel glycolipid necessary for bacitracin and daptomycin resistance in *C. difficile*.

## COMMENTARY

Organisms living in variable environments need the ability to exploit beneficial conditions as well as to endure detrimental ones. Bacteria have evolved multiple sensors that assess the external environment as well as effectors that govern adaptive changes to motility, metabolism, and gene expression in response to environmental fluctuations. Multiple conserved families of sensor and effector proteins have been identified as the components of bacterial signal transduction networks. Many of these proteins are widespread in both prokaryotes and eukaryotes. All kingdoms of life regulate cellular processes through networks of kinases and phosphatases that phosphorylate and dephosphorylate serine, threonine, or tyrosine residues on target proteins (1). Prokaryotes and eukaryotes both employ second messenger signaling, in which an extracellular signal triggers the synthesis or degradation of a small, rapidly diffusible signaling molecule—often, a modified nucleotide—within the cytoplasm (2). Other signaling mechanisms appear to be rare in eukaryotes and are attractive candidates for antibiotic development, as the lack of conserved targets in host cells limits the opportunity for therapies to cause unwanted side effects in host organisms (3).

Unique to bacteria, chemotaxis systems allow methyl-accepting chemotaxis protein (MCP) receptors to perceive signals such as ligands or altered redox environments and affect the activity of motility-enabling flagellar motors via signaling cascades that include histidine kinases and phospho-accepting coupling proteins (4). Extracytoplasmic function (ECF) sigma factors are kept dormant by interaction with anti-sigma factors in most conditions but are de-repressed by a variety of signals such as cell envelope stress (5). When activated, ECF divert RNA polymerase away from the promoters of constitutively expressed “housekeeping” genes and focus transcriptional activity on subsets of genes involved in the cellular response to the activating stimulus (5). Quorum sensing (QS) enables communication between bacterial cells, which secrete an autoinducing compound or peptide as well as a cell-surface receptor that senses it (6). Because these autoinducers are small and rapidly diffuse away from their cell of origin, an individual cell will realistically only perceive an autoinducer if it is surrounded by other cells also producing it. As some bacterial processes, such as virulence factor production, are most advantageous when done collectively, this allows bacteria to repress some processes in conditions of low cell density and activate them once they sense the presence of a supportive cohort (6).

Two-component signaling (TCS) is also a characteristic of bacteria. The two components of TCS modules are the histidine kinase (HK) and the response regulator (RR) containing a receiver domain. Histidine kinases contain sensor domains and catalytic domains that typically hydrolyze adenosine triphosphate (ATP) molecules to autophosphorylate a histidine residue and then transfer the phosphate group to an aspartic acid residue on a cognate receiver domain, although some HKs primarily act to desphosphorylate a target aspartate instead (7, 8). The cellular function of the RR protein is either activated or repressed upon phosphorylation (7, 9). Bacterial genomes often include many genes for histidine kinase and receiver domains (10). The encoded proteins exhibit high selectivity for each other, interacting with their partner proteins with affinities several orders of magnitude higher compared to non-cognate proteins from the same organism (10). However, a given histidine kinase may have a single high-affinity receiver domain or have multiple possible targets; similarly, a receiver domain may preferentially interact with a single histidine kinase or accept input from a larger subset of the histidine kinases present in the cell (7).

Response regulators have a number of cellular functions. The majority of annotated receiver domains are coupled to DNA binding domains, allowing them to serve as transcription factors, but RR output domains also include RNA binding proteins, protein-protein interaction domains, and enzymes (9). Some response regulators influence the activity of kinases, phosphatases, and second messenger synthases and hydrolases, allowing integration of multiple signaling pathways (9).

Just as receiver domains can be coupled with multiple families of effector domain to affect a wide range of cellular processes, HKs have been found coupled to multiple families of sensor domains, which can be extracellular, periplasmic, or cytoplasmic (8). Many of these sensory domain families bind a staggering variety of ligands, while others bind co-factors that render them sensitive to changes in oxygen or redox state, allowing TCS systems to convey information about diverse stimuli (8). Modern genomic and bioinformatic analytic tools make it quite easy to identify the number of TCS genes present in a genome, but it is currently impossible to predict the stimuli that regulate their activity.

Signal transduction in the spore-forming human intestinal pathogen *Clostridioides* (formerly *Clostridium*) *difficile* has been the subject of much scientific interest since the early 21st century. Between 2003 and 2005, the public health impact of this bacterium, previously identified as an uncommon cause of antibiotic-associated diarrhea, escalated quickly (11). This was due to a combination of genetic and environmental factors. Modern epidemic *C. difficile* strains emerged, capable of causing infections with more severe symptoms and higher mortality rates than those caused by historical strains (11). Increased use of clindamycin, cephalosporin, and fluoroquinolone antibiotics predisposed patients for *C. difficile* infection (CDI), as *C. difficile* is highly resilient against these classes of antibiotic (11). *C. difficile* colonization of new hosts is impeded by robust, diverse microbial communities in the mammalian gut (11). By negatively impacting the density and species diversity of the commensal microbiota, antibiotic usage pre-disposes patients for CDI by eliminating their competition for nutrients and habitats within the mammalian gut (12).

In order to colonize hosts and establish symptomatic infections*, C. difficile* cells must survive a highly variable environment. The mammalian innate immune system responds to *C. difficile* with an inflammatory immune response which attacks the pathogen with stresses such as antimicrobial peptides and reactive oxygen and reactive nitrogen species (13). In addition, antibiotics such as metronidazole, vancomycin, or fidaxomicin attack cellular processes including nucleic acid synthesis, cell wall synthesis, and transcription (14).

While the general consensus is that anaerobic bacteria encode fewer signaling proteins than their aerobic counterparts, *C. difficile* devotes a large portion of its genome to sensory and signaling proteins (15). Some sensory mechanisms appear to have been more advantageous to this organism than others. Out of over 4,000 reading frames in the CD630 genome, only one MCP and three ECF sigma factors have been identified, none of which have identified activating signals (16). Additionally, only five serine/threonine/tyrosine kinases have been identified (1). Much of *C. difficile* signaling is mediated by nucleotide second messengers. Thirty-seven genes encode synthetases or phosphodiesterases of cyclic diguanylate, which primarily affects gene transcription through an extensive network of riboswitches (17). There are four genes for metabolism of cyclic diadenylate, which has three identified protein effectors to date (18). Two genes encode synthetases for hyperphosphorylated guanine alarmones, which affect antibiotic susceptibility through unknown mechanisms (19). CD630 also encodes 44 TCS histidine kinases and 54 response regulator genes; all of these are conserved in the modern epidemic strain R20291, which encodes two additional kinases.

Of the dozens of predicted clostridial TCS, very few have been identified as regulating specific pathways or processes and even fewer have known activating signals. Some of these TCS have homologs with known functions in *Bacillus subtilis* or *Staphylococcus aureus*, allowing predictions about their biological roles, but many do not. Nine clostridial HKs and nine RRs have been linked to specific cellular processes, and an activating signal has been identified for only one of them. Many of these were initially identified in transposon libraries or mutant strains and involve defects or disregulation in cell wall synthesis or maintenance that affect cell morphology, which allowed their identification by microscopy.

The WalRK TCS consists of the bifunctional kinase/phosphatase, WalK, and its cognate RR WalR, a transcriptional regulator that is active when phosphorylated (20). The WalRK operon is broadly conserved across Firmicutes species and has been found to be essential in all species investigated to date (20). The set of genes regulated by WalRK varies from a dozen to over 100 (20). In many species, WalRK is essential in maintaining cell wall morphology (20). *C. difficile* cells are rod-shaped bacilli, but when *walRK* transcription is disrupted, the cells elongate and curve. These cells also display increased susceptibility to cell-wall active antibiotics, including ampicillin, vancomycin, and daptomycin (20). The *C. difficile walRK* operon is unique in that it also contains *walA*, which encodes a lipoprotein with no known homologs outside of *C. difficile* that is believed to modulate WalK activity (20). The clostridial *walK* gene is also unusual, as it is missing a sensory domain from the Per-Arnt-Syn (PAS) family present in *walK* from the model Gram-positive bacterium *B. subtilis*, raising questions about how WalK activity is stimulated (20).

The clostridial HexRK TCS also contributes to daptomycin resistance. HexR regulates the expression of a three-gene operon, *hexSDF,* which contributes to cell envelope homeostasis (21). *hexS* encodes a predicted monogalactosyldiacylglycerol synthase, *hexD* encodes a predicted polysaccharide deacetylase, and *hexF* encodes a putative MprF-like flippase (21). The cell membrane of *C. difficile* is highly enriched in glycolipids compared to those of model organisms, including aminohexosyl-hexosyldiradylglycerol (HNHDRG), which thus far appears to be unique to this species and ordinarily comprises approximately 16% of *C. difficile* membrane lipids (22). Pannullo and colleagues report that when *hexRK* is knocked down or knocked out, expression of *hexSDF* decreases (21). When *hexSDF* is knocked down or knocked out, HNHDRG is absent from the clostridial membrane and daptomycin resistance decreases, suggesting that *hexSDF*-mediated HNHDRG synthesis contributes to daptomycin resistance (21).

Another TCS contributing to clostridial antibiotic resistance is CprK and CprR. CprK is part of the *cprABCK* locus, along with *cprA*, *cprB,* and *cprC* which encode ABC transporter genes (23). CprK is activated by binding some types of lantibiotic and phosphorylates CprR, which then positively regulates transcription of the operon to generate the ABC transporter that exports multiple types of lantibiotic out of the cell to provide broad-spectrum lantibiotic resistance (24). CprK appears to dephosphorylate CprR in the absence of activating lantibtioics (24). The clostridial CprK appears to respond only to a subset of lantibiotics that contain certain functional groups (24). In many previously studied lantibiotic-sensing TCS systems, the response regulator and histidine kinase are co-transcribed in a single operon, and the regulated transporter is specific for one lantibiotic. However, *C. difficile* diverges from this paradigm. In *C. difficile,* the *cprR* and *cprK* genes are not linked or co-regulated, which allows differential expression of the genes (23). Both genes are expressed at a low basal level in the absence of an activating lantibiotic; lantibiotic exposure stimulates CprK kinase activity and CprR transcriptional regulation, which increase expression of *cprK* and *cprABC* but not *cprR*, leading to a stoichiometric excess of CprK compared to CprR. As CprK dephosphorylates CprR when lantibiotic stress is removed, this genetic decoupling allows faster deactivation of CprR than could be achieved by co-transcription in one operon, which would cause roughly equal amounts of the two proteins to be produced (24).

Processes other than cell envelope homeostasis and antibiotic resistance have been linked to TCS activity in *C. difficile.* The Spo0A response regulator is an “orphan” RR. In the model organism *B. subtilis, spo0A* is activated by a multi-kinase phosphorelay and is a key regulator of genes for sporulation as well as efflux pump production and metabolism (25). Mutation or deletion of *spo0A* results in an asporogenous phenotype in all species studied (26). These non-sporulating mutants also have elevated toxin production (26). The activating kinase(s) that phosphorylate Spo0A in *C. difficile* are unknown; however, the orphan HKs PtpA, PtpB, and PtpC each repress sporulation by de-phosphorylating Spo0A (27). In addition, another orphan RR, known as CD1688 in CD630 and as CDR1586 in strain R20291, regulates the transcription of several sporulation-specific genes in a phosphorylation-dependent manner and represses sporulation (28). PtpA, PtpB, PtpC, and CD1688 affect sporulation but not toxin production, revealing how targeted to specific cellular processes TCS activity can be.

TCS activity can also affect cell morphology and motility. Transcription of the *cmrRST* operon, which encodes the HK CmrS and two RRs, CmrR and CmrT, is subject to multiple levels of regulation. The operon has four transcriptional start sites, two of which are impacted by the recombinase-dependent reversible inversion of a “switch” sequence within the promoter region, introducing stochastic phase variation within the population (29, 30). In addition, an upstream cyclic diguanylate-binding riboswitch links *cmrRST* transcription to second messenger signaling (30). The switch’s “ON” orientation manifests in rough colony morphology and diminished swimming motility, while the “OFF” cells have smooth colony morphology and greater motility (29). Overexpression of CmrR or CmrT biases the population toward the rough morphology; phosphorylation of these RRs appears to allow their activity at lower concentrations but to be unneeded at high concentrations (29). CmrR and CmrT inversely regulate swimming motility and migration over solid surfaces, although they do not impact production of either flagella or type-IV pili and the mechanisms by which they impact motility are unknown (29). CmrR also autoregulates transcription of the *cmrRST* operon and is necessary for full virulence in animal models through unknown mechanisms (29, 30).

Flagellum and toxin production is affected by the AgrC HK and the AgrA RR, present in the *agr2* locus (31). All *C. difficile* strains have an *agr1* locus, which encodes only *argD* and *argB*, a QS autoinducer and the protease that processes it for export, respectively (32). Many epidemic strains have an additional *agr2* locus which encodes additional copies of *argD* and *argB* as well as the TCS genes *argC* and *argA* (32). Disruption of *C. difficile agrA* in strains that encode it decreases the transcription of genes for toxin production, flagellar biosynthesis, and cyclic diguanylate signaling (31, 32). This larger operon is homologous to that encoding the Agr operon in *Staphylococcus aureus*, in which ArgC is the sensor that perceived the secreted autoinducer and phosphorylation of AgrA is its quorum-regulated output, which autoregulates *agr* transcription as well as regulating other genes (33). It has recently been reported in a pre-print that the RgaS and RgaR TCS, comprising an orphan HK and RR rather than an operon, regulates expression of the *C. difficile agr1* locus (34). The RgaS HK is not regulated by the AgrD autoinducer, and its activating signal is unknown (34). Having this locus regulated by two differentially encoded TCS genes rather than including the TCS genes in the *agr1* locus provides opportunities for multiple signals to affect the output of the QS system.

In this work, Pannullo and colleagues have characterized an additional clostridial TCS, the DraRS system. DraS has a conserved cytoplasmic HK domain but its extracellular sensor domain has no known homologs. DraR contains a receiver domain and a DNA-binding domain, allowing it to serve as a transcription factor. This work originated in a study of cell envelope stress responses using lysozyme as a probe. The unusual glycolipid-rich cell envelope of *C. difficile* endows the bacterium with a high baseline resistance to lysozyme due to the large amount of deacetylated peptidoglycan in the cell envelope. In the presence of lysozyme, the ECF σ^V^ upregulates transcription of *pdaV,* a polysaccharide deacetylase which acts upon peptidoglycan, enhancing lysozyme resistance even further. To prevent this response from obscuring evidence of other lysozyme-induced stress responses, experiments were conducted in a *ΔcsfV-operon* strain, which lacks the σ^V^ gene and the PdaV-mediated inducible lysozyme stress response.

Increased lysozyme resistance was discovered in the draS^605*^ strain, in which a premature stop codon has excised the catalytic portion of DraS (35). Deletion of DraR, the cognate RR of DraS, restored wild-type lysozyme susceptibility in the draS^605*^ strain, suggesting that increased lysozyme resistance was due to an excess of active DraR rather than its absence. This indicated that DraS might function mainly as a phosphatase for DraR, such that deletion of DraS catalytic activity could lead to an excess of phosphorylated DraR (35). The authors confirmed this by mutating the phospho-accepting aspartic acid residue of DraR into a glutamic acid—which, as it is longer than an aspartic acid and still contains a negative charge at the end, is a structural mimic of phosphoaspartate and can serve to constitutively activate an RR. Induction of the DraR^D54E^ protein increased lysozyme resistance and upregulated *draR* transcription in a luciferase transcriptional reporter strain (35). Overproduction of wild-type DraR had no effect, confirming that phosphorylation is necessary for DraR activity. The authors used their *draR* luciferase reporter to monitor the transcriptional response of *draRS* to cell envelope stresses and found that lysozyme, as well as a suite of cell-wall active antibiotics, had no effect but that a subset of antibiotics (vancomycin, bacitracin, ramoplanin, and daptomycin), which each bind lipid-II, did upregulate *draRS* transcription (35). Notably, the lipid-II binding antibiotic nisin had no effect, so *draRS* does not respond broadly to all lipid-II binding compounds (35). The authors tested several *C. difficile* strains and found that most, but not all, increased *draRS* transcription in response to daptomycin exposure (35).

The authors then used a Δ*draRS* strain and the inducible DraR^D54E^ strain to assess how both lack and excess of DraR activity affected susceptibility to cell-envelope-active antibiotics. Although the Δ*draRS* strain did not show decreased bacitracin or daptomycin resistance, DraR^D54E^ increased bacitracin and daptomycin resistance in wild-type and Δ*draRS* backgrounds (35).

To provide a mechanism for their findings, the authors compared the transcripts of wild-type and Δ*draRS* strains in the presence and absence of daptomycin stress. The authors found that the transcriptional response to daptomycin is complex and likely involves multiple regulators beyond DraR, as many of the daptomycin-responsive genes were DraRS independent. However, 11 genes were identified as a daptomycin-activated DraR regulon, including operons for a TCS and an ABC transporter (35). Interestingly, transcriptional comparisons probing the gain of function provided by the DraR^D54E^ overexpression strain revealed a broader regulon than that provided by comparing the wild-type and Δ*draRS* strains. These 28 genes included 10 of the 11 previously identified genes as well as the *hexRK* and *hexSDF* operons as well as several genes predicted to play roles in cell wall biogenesis (35).

To explore the intersection of multiple TCS responsive to cell envelope stress, the authors induced DraR^D54E^ overexpression in wild-type *C. difficile* as well as strains with the *hexRK* and *hexSDF* operons deleted. The absence of HexRK did not mitigate the ability of DraR^D54E^ overexpression to increase daptomycin or bacitracin resistance, but in the absence of HexSDF, the effect of DraR^D54E^ on resistance to these antibiotics is dramatically reduced (35).

These authors had previously established that HexRK regulates transcription of *hexSDF* and that HexSDF-mediated HNHDRG is necessary for daptomycin resistance, although daptomycin did not stimulate *hexS* transcription under the conditions studied (21). This work identifies the activating signal of DraRS and positions DraRS as a TCS upstream of the HexRK TCS, establishing a chain of causation from daptomycin exposure to active DraR, to transcribed and activated HexR, to transcribed and activated HexSDF, to HNHDRG production, and ultimately to daptomycin resistance (21, 35). However, the map of this pathway contains many blank spaces which must contain additional sensors and regulators as yet unknown. Many of the daptomycin-regulated genes in *C. difficile* are regulated in the Δ*draRS* strain, and deletion of the *hexRK* locus does not abolish DraR^D54E^-dependent daptomycin resistance. Notably, deletion or overexpression of several genes in the daptomycin-responsive DraRS regulon had no impact on daptomycin or bacitracin resistance, so it is currently unclear how it benefited the organism to link their transcription to perception of cell envelope stress. Clearly, the clostridial response to cell envelope stress is multi-factorial and integrates the outputs of several signaling pathways. One intriguing question for future studies is the identity of the kinase that phosphorylates DraR, as DraS appears to function mainly as a phosphatase for DraR under the conditions studied. The authors suggest that DraR could be “inappropriately” phosphorylated by another protein or high-energy phosphodonor, but it is also possible that DraR is the cognate RR for one of the uncharacterized orphan HKs in the *C. difficile* genome which works in opposition to DraS.

It is striking that many of the TCS operons whose roles in *C. difficile* have been elucidated exhibit low conservation with the operons that encode homologous systems in other organisms. The clostridial *walRK* operon has acquired the unique *walA* gene. The *cprR* and *cprK* genes have been orphaned for independent transcription. Similarly, *rgaS* and *rgaR* have separated from each other and from the QS elements of the *agr1* locus. The phosphorelay that activates Spo0A in *B. subtilis* has no known clostridial homologs, although several repressors have been identified. Here, the authors note that despite low sequence homology, the clostridial DraRS TCS appears to occupy a similar functional niche as the LiaRS TCS, which mediates resistance to antibiotics active at the cell envelope in many Firmicutes. However, LiaRS is broadly activated by diverse antibiotics, while DraRS is highly specific for a subset of the antibiotics known to have lipid-II interactions. The authors especially note that DraRS is not responsive to nisin, which does activate most known homologs of LiaRS and speculate that the presence of the nisin-responsive *cpr* locus may have abrogated the need for DraRS to respond to nisin in *C. difficile* (24, 35). The *C. difficile* TCS signaling network appears to be decentralized, with many elements or roles that are co-transcribed or shared in homologous systems separated to allow more precise regulation. With dozens of clostridial TCS systems remaining to be characterized, a complete map of two-component signaling in this organism will be complex indeed.
