# Correction for Berumen Alvarez and Purcell, “Expanding our grasp of two-component signaling in *Clostridioides difficile*”

**DOI:** 10.1128/jb.00388-23

**Published:** 2023-12-04

**Authors:** Orlando Berumen Alvarez, Erin B. Purcell

## AUTHOR CORRECTION

Volume 205, no. 10, e00188-23, 2023, https://doi.org/10.1128/jb.00188-23. The article should include an Acknowledgments section consisting of the following statement: “Orlando Berumen Alvarez was supported by the National Science Foundation under grant no. CHE 2213353.”

